# The Orexigenic Effect of Ghrelin Is Mediated through Central Activation of the Endogenous Cannabinoid System

**DOI:** 10.1371/journal.pone.0001797

**Published:** 2008-03-12

**Authors:** Blerina Kola, Imre Farkas, Mirjam Christ-Crain, Gábor Wittmann, Francesca Lolli, Faisal Amin, Judith Harvey-White, Zsolt Liposits, George Kunos, Ashley B. Grossman, Csaba Fekete, Márta Korbonits

**Affiliations:** 1 Department of Endocrinology, Barts and the London School of Medicine and Dentistry, University of London, United Kingdom; 2 Department of Endocrine Neurobiology, Institute of Experimental Medicine, Hungarian Academy of Sciences, Budapest, Hungary; 3 Laboratory of Physiologic Studies, National Institute on Alcohol Abuse and Alcoholism, National Institutes of Health, Bethesda, Maryland, United States of America; 4 Tupper Research Institute, Division of Endocrinology, Diabetes, Metabolism and Molecular Medicine, Department of Medicine, Tufts-New England Medical Center, Boston, Massachusetts, United States of America; University of Parma, Italy

## Abstract

**Introduction:**

Ghrelin and cannabinoids stimulate appetite, this effect possibly being mediated by the activation of hypothalamic AMP-activated protein kinase (AMPK), a key enzyme in appetite and metabolism regulation. The cannabinoid receptor type 1 (CB1) antagonist rimonabant can block the orexigenic effect of ghrelin. In this study, we have elucidated the mechanism of the putative ghrelin-cannabinoid interaction.

**Methods:**

The effects of ghrelin and CB1 antagonist rimonabant in wild-type mice, and the effect of ghrelin in CB1-knockout animals, were studied on food intake, hypothalamic AMPK activity and endogenous cannabinoid content. In patch-clamp electrophysiology experiments the effect of ghrelin was assessed on the synaptic inputs in parvocellular neurons of the hypothalamic paraventricular nucleus, with or without the pre-administration of a CB1 antagonist or of cannabinoid synthesis inhibitors.

**Results and Conclusions:**

Ghrelin did not induce an orexigenic effect in CB1-knockout mice. Correspondingly, both the genetic lack of CB1 and the pharmacological blockade of CB1 inhibited the effect of ghrelin on AMPK activity. Ghrelin increased the endocannabinoid content of the hypothalamus in wild-type mice and this effect was abolished by rimonabant pre-treatment, while no effect was observed in CB1-KO animals. Electrophysiology studies showed that ghrelin can inhibit the excitatory inputs on the parvocellular neurons of the paraventricular nucleus, and that this effect is abolished by administration of a CB1 antagonist or an inhibitor of the DAG lipase, the enzyme responsible for 2-AG synthesis. The effect is also lost in the presence of BAPTA, an intracellular calcium chelator, which inhibits endocannabinoid synthesis in the recorded parvocellular neuron and therefore blocks the retrograde signaling exerted by endocannabinoids. In summary, an intact cannabinoid signaling pathway is necessary for the stimulatory effects of ghrelin on AMPK activity and food intake, and for the inhibitory effect of ghrelin on paraventricular neurons.

## Introduction

Ghrelin is a brain-gut peptide that stimulates appetite and also has direct effects on the regulation of energy balance in the periphery [1]. It promotes appetite via effects in the hypothalamic arcuate and paraventricular (PVN) nuclei, both known to be involved in appetite regulation [2]. Ghrelin stimulates the orexigenic neuropeptide Y/agouti-related protein neurons and inhibits the anorexigenic pro-opiomelanocortin/cocaine- and amphetamine-regulated transcript neurons, thus ultimately enhancing appetite [3], [4]. In addition, intranuclear injection of ghrelin into the PVN, where ghrelin receptor-expressing cells are present [5], also increases appetite [6]. At least one mediator of the orexigenic effect of ghrelin is AMP-activated protein kinase (AMPK) [7], [8]. AMPK is a key enzyme regulator of energy homeostasis both centrally and peripherally [2], [9]. Hypothalamic AMPK is a mediator of several appetite-regulating hormones; it is inhibited by leptin and α-melanocyte stimulating hormone and activated by ghrelin and cannabinoids [7], [8], [10]. A large body of evidence points to the role of the cannabinoid system in the hypothalamic neuronal regulation of appetite and body weight: tetrahydrocannabinol (THC), a plant-derived cannabinoid, and the endogenous cannabinoids anandamide (AEA) and 2-arachydinoyl glycerol (2-AG), have been shown to increase food intake via a specific receptor, CB1 [11]–[13]. We have recently shown that CB1-immunoreactive axons densely innervate all feeding-related nuclei in the hypothalamus, via both excitatory and inhibitory synapses [14]. CB1 is mainly localized to presynaptic axon terminals and activated by endocannabinoids synthesized and released by the postsynaptic neurons, a phenomenon otherwise known as retrograde signaling [12].

We have recently demonstrated that there is an interaction between ghrelin and cannabinoid-related actions, as sub-anorectic doses of rimonabant can inhibit the orexigenic effect of ghrelin injected focally into the PVN [6]. The corticotropin-releasing hormone- and thyrotropin-releasing hormone-secreting parvocellular neurons of the PVN are known to have an inhibitory effect on food intake [4]. In the current study we have hypothesized that the appetite-inducing effects of ghrelin are mediated by the endogenous cannabinoid system. We have investigated the interaction between ghrelin and the cannabinoid systems on the mechanisms underlying appetite regulation by *in vivo* studies using rimonabant, a known antagonist of CB1, and by a genetic approach using CB1-knockout (CB1-KO) mice; we have also utilized an *in vitro* electrophysiological system to study the interaction of the two systems on parvocellular neurons of the PVN of mice. We show here that ghrelin does not induce appetite in CB1-KO mice. Furthermore, while ghrelin stimulates hypothalamic AMPK activity in wild-type mice, it has no effect on AMPK in CB1-KO or in CB1 antagonist-treated mice. The electrophysiological studies show that ghrelin can inhibit the excitatory inputs in the parvocellular neurons of the PVN and that this effect can be abolished by administration of a CB1 antagonist or THL, an inhibitor of the 2-AG synthesizing enzyme DAG lipase. The effect is also lost in the presence of BAPTA, an intracellular calcium chelator, which inhibits the endocannabinoid synthesis in the recorded cell and therefore blocks retrograde signaling exerted by endocannabinoids. Thus, the effect of ghrelin on hypothalamic AMPK, on neuronal activity in the PVN and ultimately on appetite, are dependent on CB1, and these data would be compatible with the existence of a ghrelin→endocannabinoid→CB1→AMPK→appetite signaling cascade.

## Results

### A) Food intake

**Figure 1:**
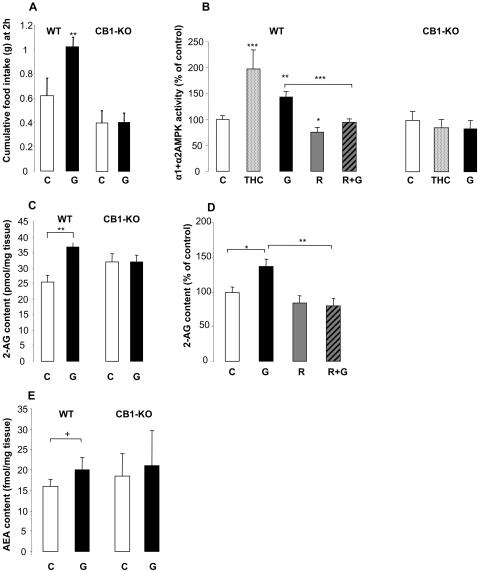
The effect of ghrelin and cannabinoids on food intake, hypothalamic AMPK activity and endocannabinoid content. (A) Cumulative food intake of WT and CB1-KO mice after 2 hours of treatment with icv ghrelin (G) or vehicle (C), n = 5–9 mice/group. (B) Ghrelin and cannabinoid effects on hypothalamic AMPK activity one hour after intraperitoneal administration of THC, ghrelin, rimonabant (R) or a combination of rimonabant and ghrelin (R+G) in WT and CB1-KO animals compared to vehicle-treated animals, n = 6–14 mice/group. (C) Hypothalamic 2-AG content after intraperitoneal treatment with ghrelin in WT and CB1-KO animals, n = 6 mice/group. (D) Hypothalamic 2-AG content in WT animals after ip treatment with ghrelin and rimonabant compared to vehicle treated animals, n = 6–14 mice/group. (E) Hypothalamic AEA content after ip treatment with ghrelin in WT and CB1-KO animals. All data shown as mean±SEM, *P<0.05, **P<0.01, ***P<0.001, ^+^P = 0.052.

Central administration of 1 µg ghrelin significantly increased the 2 h food intake in wild-type (WT) animals (ghrelin vs. control in WT mice: 1.02±0.08 g vs. 0.62±0.15 g Kruskal-Wallis test: n = 5–9, df = 3, overall T = 14.4, overall P = 0.0024; for individual comparison [Conover-Inman test]: critical t (21 df) = 2.07, ghrelin vs. control P = 0.0045, Fig. 1A). In contrast, ghrelin had no effect on food intake in the CB1-KO mice (ghrelin vs. control in CB1-KO mice: 0.42±0.07 g vs. 0.41±0.1 g, P = 0.7), suggesting that the effect of ghrelin on appetite is dependent on CB1.

### B) AMPK activity in WT and CB1-KO mice

Both THC and ghrelin significantly increased AMPK activity in the hypothalamus of WT mice (THC: 197±40% of control, ghrelin: 143.2±11% of control, Kruskal-Wallis test: n = 6–14, df = 4, overall T = 23.64, overall P<0.0001; Conover-Inman test critical t (45 df) = 2.014, THC vs. control P = 0.001; ghrelin vs. control P = 0.0038; Fig. 1B), while rimonabant significantly decreased basal AMPK activity (75.7±8.4% of control, P = 0.0245). Rimonabant co-administration with ghrelin inhibited the stimulatory effect of ghrelin: AMPK activity levels in this group were similar to the control group (94.7±6.9% of control), and significantly lower than that in the animals treated with ghrelin alone (143.2±11% of control, P = 0.0008). In CB1-KO animals, THC did not modulate hypothalamic AMPK activity (85±16% of control, Fig. 1B), suggesting that the AMPK-stimulating effect of THC is via CB1. Ghrelin administration also had no effect on AMPK activity in CB1-KO animals (87.7±18.5%, Fig. 1B), suggesting that the AMPK-stimulating effect of ghrelin is CB1-dependent.

### C) Endocannabinoid content of the hypothalamus

Ghrelin treatment significantly increased the 2-AG content of the hypothalamus of WT mice (control 25.67±2.21 vs. ghrelin 36.6±1.59 pmol/mg tissue, Student's unpaired t test, n = 6, df = 9, t = 3.519, P = 0.0065, Fig. 1C). Rimonabant co-administration with ghrelin prevented the ghrelin-induced increase in 2-AG levels (79.8±10.2% of control, Kruskal-Wallis test, n = 6–14, df = 3, overall T = 12.62, overall P = 0.0055; Conover-Inman test critical t (36 df) = 2.028, P = 0.0015 vs. ghrelin, Fig. 1D). In CB1-KO mice, ghrelin did not change 2-AG content (control 32.0±2.6 vs. ghrelin 32.2±2.2 pmol/mg, Fig. 1C). Anandamide levels showed a trend towards an increase following ghrelin treatment in WT mice (control 15.9±1.8 vs. ghrelin 19.9±3.12 fmol/mg, P = 0.052, Fig. 1E), whereas no effect was observed in CB1-KO mice (control 18.5±5.6 vs. ghrelin 20.9±8.7 fmol/mg, Fig. 1E). Rimonabant treatment did not affect AEA levels, and 1-AG levels were not influenced by any of the treatments (data not shown).

### D) Patch-clamp electrophysiology of parvocellular neurons in the PVN

**Figure 2:**
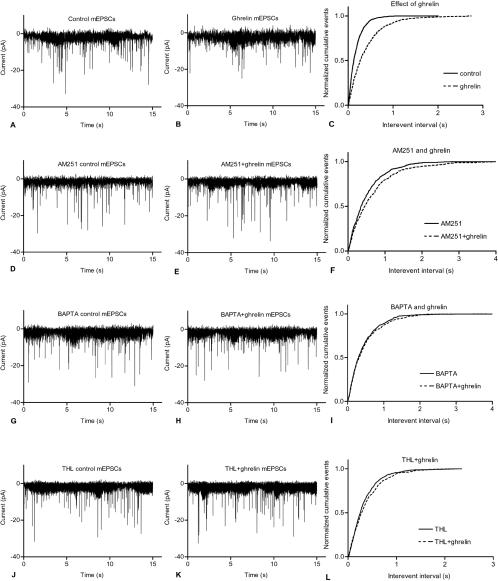
Whole-cell patch clamp recordings of mEPSCs in the parvocellular neurons of the PVN. Application of ghrelin (100 nM) in the extracellular solution decreased the amplitude and frequency of the mEPSCs (Fig. 2A–C). Extracellular administration of the cannabinoid receptor antagonist AM251 (1 µM), however, blocked the effect of ghrelin. Both amplitude and frequency changes were attenuated (Fig. 2D–F). In addition, intracellularly applied BAPTA, an effective chelator of free calcium, (Fig. 2G–I) and the DAG lipase (DAGL) inhibitor THL (5 µM) (Fig. 2J–L) also abolished the effect of ghrelin.

**Figure 3:**
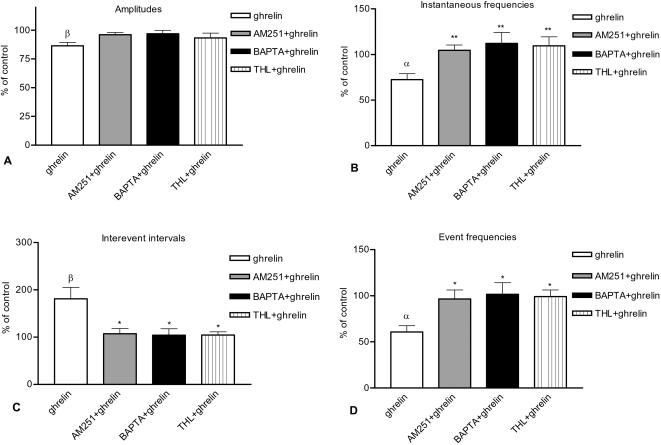
Changes of various parameters of mEPSCs [(A) amplitude, (B) instantaneous frequency, (C) interevent interval, (D) event frequency] elicited by ghrelin (as percentage of control), by AM251+ghrelin (as percentage of AM251 alone), BAPTA+ghrelin (as percentage of BAPTA alone) and THL+ghrelin (as percentage of THL alone) in the parvocellular neurons. Application of ghrelin resulted in significant changes in all of these parameters whereas administration of AM251, BAPTA or THL eliminated the changes (Fig. 3A–D). All data shown as mean±SEM, α and β correspond to P<0.05 and P<0.01, respectively, and refer to the comparison of ghrelin vs. control. *P<0.05 and **P<0.01 correspond to the comparison between ghrelin vs. AM251+ghrelin, ghrelin vs. BAPTA+ghrelin or ghrelin vs. THL+ghrelin groups.

Whole cell patch-clamp recordings of the parvocellular neurons of the PVN were used to characterize the postsynaptic currents and the modulatory effect of ghrelin. The recorded inward current pulses were miniature excitatory postsynaptic currents (mEPSCs) as all pulses were abolished by the extracellularly applied kynurenic acid (5 mM), a non-selective antagonist of the glutamate receptor (data not shown). Ghrelin (100 nM) significantly inhibited the mEPSCs of PVN neurons. Ghrelin decreased the amplitude of the pulses to 86.46±2.86% of the control (control −13.89±0.87pA, ghrelin −11.96±0.62pA; Student's paired t-test n = 8, df = 7, P = 0.0076), and the instantaneous frequency to 72.38±6.57% of the control (control 11.69±1.67Hz, ghrelin 7.79±0.53Hz; Student's paired t-test n = 8, df = 7, P = 0.0192). Ghrelin also increased the interevent interval to 181.6±24.1% of the control (control 332±79.2 ms, ghrelin 507±71.4 ms; Student's paired t-test n = 8, df = 7, P = 0.0003). Ghrelin treatment decreased the event frequency to 60.6±6.95% of the control (control 4.26±0.96Hz, ghrelin 2.21±0.26Hz; Student's paired t-test n = 8, df = 7, P = 0.0320) (Fig. 2A–B and Fig. 3A–D). The distribution of the normalized cumulative events also showed changes (Fig. 2C), demonstrating that ghrelin modified the synaptic excitatory transmission of the parvocellular neurons of the PVN.

In order to examine the involvement of CB1 in mediating the effect of ghrelin, the CB1 antagonist AM251 (1 µM) was added to the extracellular solution and 15 min later ghrelin was administered (100 nM). Application of AM251 eliminated the changes caused by ghrelin, since none of the four parameters analyzed differed significantly from the values calculated from the recordings performed without ghrelin (Fig. 2D–E and Fig. 3A–D). Amplitude was 98±2.09% of the AM251 control recordings without ghrelin (AM251 −16.47±1.60pA, AM251+ghrelin −15.89±1.80pA), instantaneous frequency was 104.6±5.72% of the AM251 control (AM251 9.04±1.21Hz, AM251+ghrelin 9.47±1.41Hz), interevent interval was 107.8±11.98% of the AM251 control (AM251 372±85.9 ms, AM251+ghrelin 339±54.7 ms), event frequency was 96.4±9.91% of the AM251 control (AM251 3.28±0.71Hz, AM251+ghrelin 3.32±0.64Hz; n = 9). The cumulative event distribution also showed an attenuation of the effect of ghrelin (Fig. 2F), indicating that CB1 was involved in the signal evoked by ghrelin.

In order to determine whether the recorded PVN neurons were involved in the ghrelin-induced production of endocannabinoids acting on CB1 of the presynaptic excitatory terminals, BAPTA was added to the intracellular solution filled into the recording electrode. The presence of BAPTA in the intracellular solution abolished the effect of ghrelin (Fig. 2G–H and Fig. 3A–D). None of the four parameters studied were different in the ghrelin-treated cells from the BAPTA control readings: average amplitude of the mEPSCs was 94.92±2.91% of the BAPTA control (BAPTA −13.89±0.886pA, BAPTA+ghrelin −11.96±0.617pA), average instantaneous frequency was 112.2±12.42% of the BAPTA control (BAPTA 5.11±0.399Hz, BAPTA+ghrelin 5.61±0.465Hz), interevent interval was 104.3±13.85% of the BAPTA control (BAPTA 625.8±58.28ms, BAPTA+ghrelin 636.4±68.66ms) and event frequency was 101.6±12.64% of the BAPTA control (BAPTA 1.65±0.169Hz, BAPTA+ghrelin 1.64±0.17Hz; n = 7). The cumulative event distribution showed a reduction in the effect of ghrelin (Fig. 2I), indicating that calcium signal transduction in the postsynaptic parvocellular neuron plays an indispensable role in the effect elicited by ghrelin.

After demonstrating that change in the calcium content of the postsynaptic cell is involved in the effect of ghrelin, the DAG lipase inhibitor THL was used to investigate the role of endocannabinoid synthesis in the mediation of the ghrelin-induced inhibition of the mEPSCs. Application of THL blocked the effect of ghrelin (Fig. 2J–L and Fig. 3A–D). The average amplitude of the mEPSCs was 92.29±4.02% of the THL control (THL−18.69±0.825pA, THL+ghrelin −17.04±0.969pA), the average instantaneous frequency was 107.4±9.95% of the THL control (THL 6.25±0.851Hz, THL+ghrelin 6.76±0.962Hz), the interevent interval was 104.3±6.79% of the THL control (THL 562.7±95.91 ms, THL+ghrelin 577.8±101.0 ms) and the event frequency was 99.14±7.11% of the THL control (THL 2.239±0.512Hz, THL+ghrelin 2.234±0.572Hz; n = 7).

We performed inter-group analysis on the parameters of mEPSC (Fig. 3A–D). Amplitudes showed no significant changes (ghrelin compared to BAPTA+ghrelin, AM251+ghrelin, or THL+ghrelin, Newman-Keuls test). The other three parameters (instantaneous frequency, interevent interval and event frequencies), however, exhibited significant differences in the ghrelin vs. BAPTA+ghrelin, the ghrelin vs. AM251+ghrelin and the ghrelin vs. THL+ghrelin analyses but not in the BAPTA+ghrelin vs. AM251+ghrelin, the BAPTA+ghrelin vs. THL+ghrelin and the AM251+ghrelin vs. THL+ghrelin analyses, suggesting that for these parameters the CB1 receptor antagonist, the 2-AG synthesis inhibitor and the intracellular Ca^2+^-chelator inhibited the effects exerted by ghrelin (Instantaneous frequency ANOVA: P = 0.0090, F = 5.003; Newman-Keuls test: ghrelin vs BAPTA+ghrelin P = 0.023, ghrelin vs. AM251+ghrelin P = 0.031, ghrelin vs. THL+ghrelin P = 0.020. Interevent interval ANOVA: P = 0.0064, F = 5.424; Newman-Keuls test: ghrelin vs BAPTA+ghrelin P = 0.017, ghrelin vs. AM251+ghrelin P = 0.0073, ghrelin vs. THL+ghrelin P = 0.012. Event frequency ANOVA: P = 0.0052, F = 5.675; Newman-Keuls test: ghrelin vs BAPTA+ghrelin P = 0.013, ghrelin vs. AM251+ghrelin P = 0.0082, ghrelin vs. THL+ghrelin P = 0.0067; df = 30).

## Discussion

In this study we have shown that the effect of ghrelin on the mechanism of appetite regulation is CB1-dependent: (i) ghrelin and cannabinoids increase hypothalamic AMPK activity and an intact CB1 receptor is mandatory for these effects; (ii) ghrelin increases the cannabinoid content of the hypothalamus and interestingly CB1 is also involved in this effect; (iii) ghrelin inhibits excitatory synaptic input in the PVN, an effect which can be abolished by a CB1 antagonist as well as via inhibition of cannabinoid synthesis with the use of BAPTA, an intracellular calcium chelator, and with the use of THL, an inhibitor of the 2-AG synthesizing enzyme DAG lipase; and ultimately (iv) ghrelin stimulates appetite and an intact CB1 receptor is necessary for this effect. These data provide evidence that an interaction between ghrelin and the cannabinoid systems is crucial for the appetite-inducing effect of ghrelin.

We have previously shown that rimonabant inhibits the orexigenic effect of ghrelin in the rat when ghrelin is administered directly into the PVN [6], and more recently chronic administration of rimonabant was also shown to suppress the orexigenic effect of the ghrelin-mimetic hexarelin [15]. As rimonabant has certain CB1 receptor-independent actions [16], [17], we used CB1-KO animals to show that the ghrelin effects involve the CB1 receptor. On the other hand, our rimonabant data in wild-type animals suggest that the lack of effect of ghrelin in CB1-KO animals is not due to compensatory mechanisms induced during embryonic development. Our study conclusively establishes the critical role of CB1 in mediating the effects of ghrelin on AMPK and appetite. We have now demonstrated that the orexigenic effect of ghrelin is absent in CB1-KO mice, substantiating the involvement of the cannabinoid system in the effects of ghrelin. Ghrelin's effects on AMPK, thought to mediate its orexigenic effects, are now also definitively shown to be CB1-dependent. Numerous studies have established that AMPK is involved in appetite regulation [10] and we have reported, for the first time, that cannabinoids stimulate hypothalamic AMPK activity while we and others have shown that ghrelin also has similar effect [7], [8]. Our present data demonstrate that the effect of ghrelin on hypothalamic AMPK activity is also CB1-dependent. Ghrelin did not affect AMPK activity in CB1-KO hypothalamus, and pre-administration of the CB1 antagonist rimonabant blocked the stimulatory effect of ghrelin on AMPK activity, indicating that the ghrelin-AMPK interaction requires an intact cannabinoid signaling system in the hypothalamus. The concordance between the effects of rimonabant and the findings in CB1-KO animals at the level of food intake, AMPK activity and cannabinoid content clearly supports our hypothesis. Therefore, the effect of ghrelin on both AMPK and appetite is clearly dependent on an intact cannabinoid pathway.

Based on these data, we suggest the possibility that ghrelin may activate CB1 by increasing the synthesis of endocannabinoids. Endogenous hypothalamic cannabinoid levels have been reported to increase with fasting and to decrease immediately after re-feeding [11], suggesting that they may play a role in determining hunger and satiety. The anorexigenic effect of leptin also seems to be mediated by reduced endocannabinoid levels [18]. In addition, glucocorticoids have also been shown to influence the endocannabinoid content of the hypothalamus and exert an inhibitory effect on glutamate release onto parvocellular neurons of the PVN via an increase in the synthesis of endogenous cannabinoids [19], [20]. The implication of the endogenous cannabinoid system on the effects of the melanocortin system is contradictory, as α-MSH, at doses which lead to inhibition of food intake, does not inhibit endocannabinoid levels, although the MC4R receptor antagonist HS014 has a late stimulatory effect on 2-AG and AEA levels [21]. Furthermore, the appetite stimulatory effect of another MC4R antagonist, JKC-363, has previously been shown to be attenuated by CB1 receptor blockade [22]. Similarly, the orexigenic effect of orexin A/hypocretin 1 is also blocked by rimonabant administration [23]. The effect of insulin on hypothalamic endocannabinoid content, at doses which would inhibit food intake, has not been reported, but insulin at lower doses which lead to inhibition of hepatic glucose output does not affect hypothalamic endocannabinoid content [21]. In this study we have shown that ghrelin also influences the hypothalamic endocannabinoid content: ghrelin significantly increased 2-AG content in the hypothalamus of WT mice, suggesting that the effect of ghrelin involves an increase in 2-AG synthesis, which then can stimulate CB1. The activated CB1 receptor then leads to an increase in AMPK activity, and we suggest ultimately an increase in appetite. Ghrelin exerts its hypothalamic effects via the growth hormone secretagogue receptor type 1a, which is a Gq-PKC pathway-coupled receptor. It has recently been suggested that other receptors using this pathway may also stimulate endocannabinoid synthesis in neuronal cells [24]. Interestingly, rimonabant administration blocked the stimulatory effect of ghrelin on 2-AG content and ghrelin had no effect on cannabinoid content of the hypothalamus in CB1-KO animals. These data suggest not only that CB1 is necessary for the effect of ghrelin on AMPK and appetite, but also that there may be a positive feedback between the CB1 and the endogenous 2-AG synthesis. This novel finding is compatible with recent data which showed increased extracellular 2-AG levels in rat hypothalamus in response to cannabinoid-agonist stimulation and decreased 2-AG levels after 10 mg/kg ip rimonabant treatment [25]. In concordance, with our data, 3 mg/kg rimonabant did not have an effect on extracellular 2-AG and AEA levels but it antagonized the effect of the CB1 agonist WIN55,212-2 on the endocannabinoid release. Bequet et al. also suggest that rimonabant doses up to 10 mg/kg do not affect hypothalamus tissue content of endocannabinoids. Interestingly in this study, AEA release was stimulated by 10 mg/kg rimonabant, suggesting a different regulation and possibly different physiological roles for AEA and 2-AG [25]. The effects of ghrelin seem to be mediated predominantly by 2-AG as this has been the endocannabinoid affected predominantly by ghrelin and our electrophysiology data with a 2-AG synthesis inhibitor THL also supports the 2-AG mediation.

The positive feed-back between CB1 receptor and endocannabinoid synthesis suggested by these data could explain the lack of effect of ghrelin in the CB1-KO. Di Marzo et al. previously showed that there is no difference in hypothalamic cannabinoid levels between WT and CB1-KO mice [26] and our data correspond to these findings. We postulate that in WT mice ghrelin would increase endocannabinoid synthesis, whose levels are then further amplified by CB1 stimulation, thus leading to further endocannabinoid biosynthesis. In this way, the presence of both ghrelin and CB1 receptors would be necessary. The effect of ghrelin on endocannabinoid synthesis could also be an indirect one through stimulation of NPY. Similarly to ghrelin, the orexigenic effect of NPY is abolished by rimonabant administration and is totally lost in CB1-KO mice [27]. Interestingly, CB1 activation stimulates NPY release [28] therefore a positive feed-back loop between endocannabinoids and ghrelin-induced NPY would partly explain the lack of effect in CB1-KO animals. Conversely, tonic CB1 receptor stimulation alone, without concomitant ghrelin receptor stimulation, necessary to trigger both NPY and endocannabinoid synthesis, might not be sufficient to increase sufficiently the endogenous 2-AG levels, and this might explain why CB1-KO mice do not exhibit lower endocannabinoid levels in the hypothalamus.

In this study, we show that the AMPK-stimulating effect of cannabinoids is also CB1-dependent as it can be blocked by rimonabant and it is absent in CB1-KO animals. These data suggest that the CB1-dependent effects of ghrelin on AMPK activity and appetite are related. The lack of an orexigenic effect of ghrelin in CB1-KO animals could contribute to their lean phenotype.

**Figure 4:**
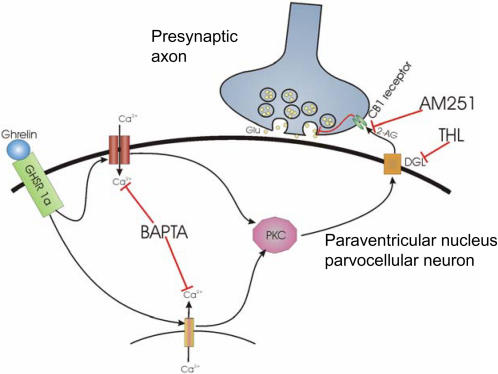
Schematic drawing illustrates the proposed model of ghrelin's action in the parvocellular neurons of the PVN. Binding of ghrelin to its receptor (growth hormone secretagogue receptor 1a, GHS-R1a) on the surface of parvocellular neurons results in an increase of intracellular Ca^2+^ levels due to mobilization of Ca^2+^ from intracellular stores and opening Ca^2+^ channels. The increased intracellular Ca^2+^ level activates the 2-AG synthesizing enzyme diacylglycerol lipase-α (DAGL), directly or through activation of protein kinase C (PKC), resulting in an increased 2-AG synthesis and release into the extracellular space. The increased activation of presynaptic CB1 then inhibits the release of the excitatory neurotransmitter glutamate (Glu) from the axons innervating the PVN neurons. Intracellular administration of the Ca^2+^-chelator BAPTA blocks this cascade by preventing the increase of intracellular Ca^2+^ level, whereas extracellularly-given THL blocks the 2-AG synthesis and AM251 blocks the cascade at the level of CB1.

Since in the hypothalamus ghrelin preferentially stimulated the synthesis of 2-AG, the endocannabinoid that primarily acts in the excitatory synapses [29], we have studied the influence of ghrelin on the excitatory input of parvocellular neurons in the PVN. We observed that ghrelin inhibits the excitatory input on PVN neurons and this effect is blocked by the co-administration of the DAG lipase inhibitor, THL or CB1 antagonist, AM251, suggesting that 2-AG synthesis and functional CB1 is required for ghrelin to result in this effect. As we have hypothesized that endocannabinoid synthesis in the recorded PVN neurons themselves may play an important role in the effect of ghrelin, and as endocannabinoid synthesis is calcium dependent, we blocked intracellular calcium by using BAPTA in the recording electrode. Extracellular ghrelin treatment together with the intracellular BAPTA administration failed to inhibit the excitatory input to the postsynaptic neurons of the PVN. This finding suggests that the blockade of endocannabinoid synthesis by BAPTA in the postsynaptic cell itself results in blockade of endocannabinoid release and subsequent lack of stimulation on the presynaptic CB1, leading to a loss of inhibition of the excitatory glutamate release from the presynaptic terminal. An outline of the suggested pathway is shown on Fig. 4. Further studies are needed to determine whether similar interaction of ghrelin and cannabinoid signaling exists in other ghrelin sensitive hypothalamic regions.

In conclusion, the effects of ghrelin investigated in this paper require an increased release of endocannabinoids, acting through CB1, to stimulate AMPK and appetite. The hypothalamic neurophysiological effects of ghrelin also require the presence of CB1. Taken together, our data indicate that the endogenous cannabinoid system is necessary for the mediation of the orexigenic and central AMPK-stimulatory effects of ghrelin.

## Materials and Methods

### Animals

The experimental CB1-KO and WT mice were derived from a genotyped stock obtained from IRIBHN, Université Libre de Bruxelles [30], and were bred at the Institute of Experimental Medicine (IEM), Budapest. The parent (Belgian) stock was generated from heterozygotes bred for 14 generations on a CD1 (Charles River, France) outbred background, with selection for the mutant CB1 gene at each generation. Adult male WT (n = 6–8) and age-matched CB1-KO littermates (n = 6) weighing 30–35g, were used in the *in vivo* experiments. For electrophysiology studies, 30±5day old CD1 WT mice were used. The animals were housed under standard environmental conditions (light between 0600–1800 h, temperature 22±1°C, rodent chow and water ad *libitum*). All experimental protocols were reviewed and approved by the Animal Welfare Committee at the IEM.

In the first set of experiments, WT and CB1-KO mice were injected intraperitoneally (ip) with 500 µg/kg THC (Tocris, Avonmouth, UK) and 500 µg/kg rat ghrelin (kind donation of Prof. Kojima, Japan) in a volume of 100 µl [31]. In the second set of experiments, WT mice were injected ip with 3 mg/kg rimonabant (SR141716; Sanofi-Aventis, Paris, France) or vehicle and 10 min later, with 500 µg/kg ghrelin or vehicle. One hour after the treatment, while no food was provided, the animals of both experiments were decapitated. Tissue samples were immediately frozen on dry ice and were stored at −80°C until assay. Hypothalami from the second experiment were processed for measurement of endogenous cannabinoid content. All injections were performed between 0900 h and 1200 h.

In a third experiment, WT and CB1-KO mice were implanted with an intracerebroventricular (icv) cannula [32]. WT and CB1-KO mice were implanted with 25-gauge stainless steel guide cannula (Small Parts Inc., Miami Lakes, FL) into the lateral cerebral ventricle under stereotactic control (coordinates from Bregma anteroventral −0.2; lateral 1.0; dorsoventral 2.0) through a burr hole in the skull [32]. The cannula was secured to the skull with ‘Crazy Glue’ (Electron Microscopy Sciences, Fort Washington, PA) and dental cement, and temporarily occluded with a dummy cannula. Bacitracin ointment was applied to the interface of the cement and the skin. Animals were weighed daily and those showing signs of illness or weight loss were removed from the study and euthanized. One week after icv cannulation, both WT and CB1-KO mice were divided into two groups and received either vehicle or 1 µg of ghrelin icv in 4 µl aCSF between 0900h and 1000h in the light phase: food intake was measured at 2 h. The mice had free access to food before and during the experiment.

### AMPK activity assay

The kinase assay for AMPK activity has been previously described [8], [33]. Briefly, hypothalami of mice were weighed and homogenized with Precellys 24 using CK14 tubes containing ceramic beads (Stretton Scientific, Stretton, UK) at 6000rpm for 1 cycle of 20 sec in lysis buffer containing 50 mM Tris-HCl, 50 mM NaF, 5 mM Na pyrophosphate, 1 mM EDTA, 250 mM sucrose, 1% Triton X-100, 1 mM DTT, 1mM benzamidine, 0.1 mM phenylmethane sulfonyl fluoride, 5 µg/ml soybean trypsin inhibitor, and the tissue protein content was determined using BCA assay (Pierce, Rockford, USA). AMPK was immunoprecipitated with an equal mixture of α1AMPK and α2AMPK antibodies [33] and AMPK activity was determined by the entity of phosphorylation of SAMS (Pepceuticals Ltd., Nottingham, UK), a synthetic peptide substrate of AMPK.

### Endocannabinoid content measurement

Following homogenization as above chloroform extraction was performed and samples dried under nitrogen. An extracted blank containing the d_4_-anandamide, but no tissue, was included each time to control if any apparent cannabinoids or other contaminants were present during the extraction procedure. Tissue levels of anandamide, 2-AG and 1-AG were quantified by liquid chromatography/in-line mass spectrophotometry, as previously described [18], [34]. The amount of anandamide, 2-AG and 1-AG in the samples was determined by using inverse linear regression of standard curves. Values were calculated as pmol or fmol per mg of wet tissue.

### Whole-cell clamp experiments

WT mice were killed by cervical dislocation and were decapitated. The brain was removed in less than 1 min, and then immersed in ice cold artificial cerebrospinal fluid (aCSF; NaCl 140 mM, KCl 3 mM, MgSO_4_ 1.3 mM, NaH_2_PO_4_ 1.4 mM, CaCl_2_ 2.4 mM, glucose 11 mM, HEPES 5 mM, pH 7.25 with NaOH) bubbled with O_2_. Hypothalamic blocks were dissected from the mouse brains and 300 µm thick slices containing the PVN were sectioned with a VT-1000S vibratome (Leica GmBH, Germany) using a sapphire knife (Delaware Diamond Knives Inc., Wilmington, DE) in ice-cold oxygenated aCSF. The slices were bisected along the third ventricle and equilibrated in aCSF saturated with O_2_ at room temperature for 1.5 h. In order to record postsynaptic currents in the neurons, the equilibrated hemi-slices were placed in an immersion-type recording chamber. The brain slices were oxygenated, during recording at RT, by bubbling the aCSF with O_2_ gas during recording at room temperature. The cells were voltage clamped at room temperature using a whole-cell clamp configuration. The instruments used for electrophysiology were as follows: Axopatch 200B patch clamp amplifier, Digidata-1322A data acquisition system and pCLAMP 9.2 software (Axon Instruments-Molecular Devices Co., Sunnyvale, CA). The headstage of the amplifier was fitted to a MHW-3 hydraulic micromanipulator (Narishige Co., Japan). The cells were visualized by a BX51WI upright microscope (Olympus Co. Japan) equipped with infrared-DIC optics and a Cohu 4912 CCD camera (Cohu Inc. San Diego, CA) driven by a Scion Image for Windows Beta 4.0.2 software (Scion Co., Frederick, MD). The microscope and the micromanipulator were fitted to an S'Table antivibration table equipped with a Petra platform (Supertech Co., Hungary-Switzerland). The patch electrodes (OD = 1.5mm, thin wall, Garner Co., U.S.A.) were pulled with a Flaming-Brown P-97 horizontal puller (Sutter Instrument Co., Novato, California, U.S.A.) and polished with an MF-830 microforge (Narishige). The resistance of the patch electrodes was 2–3 MΩ.

The intracellular pipette solution used for electrophysiological recording contained HEPES 10 mM, K-gluconate 120 mM, KCl 10 mM, NaCl 1 mM, MgCl_2_ 1 mM, EGTA 1 mM, Mg-ATP 2 mM, Na-GTP 0.3 mM, pH 7.25 with KOH, osmolarity was set to 290–295 mOsm using D-sorbitol. In order to block intracellular calcium-dependent signal transduction pathways, intracellular calcium was chelated by substituting EGTA with 10 mM BAPTA (1,2-Bis(2-aminophenoxy)ethane-*N,N,N′,N′*-tetraacetic acid) in the intracellular pipette solution. When BAPTA was used in the intracellular solution, we waited 15 min after establishing a stable whole-cell clamp configuration in order to equilibrate the intracellular matrix and the electrode solution containing BAPTA. The holding potential was −70 mV. Pipette offset potential, series resistance and capacitance were compensated before recording. Only cells with low leakage and stable baseline were used for electrophysiological measurements. The cells requiring any leak subtraction were omitted. The PVN parvocellular cells were identified in the acute brain slices by their apparent topographic location in the PVN. After establishing a stable whole-cell clamp configuration the cells were identified as neurons by evoking action potential by injecting +10pA current with −10pA prepulse in current clamp mode. In order to block voltage sensitive Na-channels and inhibitory postsynaptic currents (IPSCs) 1 µM tetrodotoxin (TTX, Tocris) and 100 µM picrotoxin (Sigma) were added to the aCSF 10 min before the start of recording of excitatory postsynaptic currents (mEPSCs). When the CB1-antagonist AM251 (1 µM, Tocris) or the DAG lipase inhibitor THL (tetrahydrolipstatin, 5 µM, Sigma) were used, they were added to the aCSF containing TTX and picrotoxin. In order to determine the effect of ghrelin, first a control measurement of the mEPSCs was carried out in a neuron. Then ghrelin (100 nM) was added to the aCSF and 15min later the mEPSCs were recorded again. Each recording lasted 258sec. In order to block excitatory inputs of the neurons examined, the glutamate receptor inhibitor kynurenic acid (5 mM, Sigma) was applied in the extracellular solution after recording control EPSCs.

In order to demonstrate the effect of ghrelin, four parameters of the mEPSCs were analyzed: **amplitude** of the pulses; the **interevent interval** representing the period between the peaks of the current and the previous event; the **instantaneous frequency** representing event frequency at the rate of the current and the previous event; and the **event frequency** representing event frequency over the entire data set (i.e. over the 258sec recording).

### Statistical analysis

Data were analyzed using the Student's t-test, the ANOVA followed by the Newman-Keuls test or the Kruskal-Wallis test followed by Conover-Inman comparison, as appropriate (GraphPad Software Inc., San Diego, CA). Electrophysiological recordings were carried out on at least 8 cells for each experimental group. Baseline correction of the mEPSC recordings was carried out using the Corrector software (L. Tatai, G. Lőcsei and B. Wittner, KOKI, Budapest). Event detection was performed using the Clampfit module of the PClamp 9.2 software (Molecular Devices, Union City, CA). Significance was taken at P<0.05. The data are expressed as mean±standard error (SEM), n = 6–8 in each treatment group, except where differently specified.
